# Synthesis and Structural and Strength Properties of xLi_2_ZrO_3_-(1-x)MgO Ceramics—Materials for Blankets

**DOI:** 10.3390/ma16145176

**Published:** 2023-07-23

**Authors:** Dmitriy I. Shlimas, Daryn B. Borgekov, Artem L. Kozlovskiy, Maxim V. Zdorovets

**Affiliations:** 1 Laboratory of Solid State Physics, The Institute of Nuclear Physics, Almaty 050032, Kazakhstan; 2 Engineering Profile Laboratory, L.N. Gumilyov Eurasian National University, Astana 010008, Kazakhstan; 3 Department of General Physics, Satbayev University, Almaty 050032, Kazakhstan

**Keywords:** lithium ceramics, blankets, doping, stability, strength properties

## Abstract

The article considers the effect of doping with magnesium oxide (MgO) on changes in the properties of lithium-containing ceramics based on lithium metazirconate (Li_2_ZrO_3_). There is interest in this type of ceramics on account of their prospects for application in tritium production in thermonuclear power engineering, as well as several other applications related to alternative energy sources. During the investigations undertaken, it was found that variation in the MgO dopant concentration above 0.10–0.15 mol resulted in the formation of impurity inclusions in the ceramic structure in the form of a MgLi_2_ZrO_4_ phase, the presence of which resulted in a rise in the density of the ceramics, along with elevation in resistance to external influences. Moreover, during experimental work on the study of the thermal stability of the ceramics to external influences, it was found that the formation of two-phase ceramics resulted in growth in the preservation of stable strength properties during high-temperature cyclic tests. The decrease in strength characteristics was observed to be less than 1%.

## 1. Introduction

The rapid development of industry and the increase in resource and energy consumption poses great risks for humanity in the near future in terms of shortage of energy resources and the consequences of their use [1,2,3]. However, despite the growing demand for energy, there is much focus on work to reduce the dependence on hydrocarbons and traditional fossil energy sources. To do this, it is proposed to use various alternative energy sources, with particular emphasis on nuclear and thermonuclear energy. In the case of nuclear energy, in the past few years, the emphasis has been on a transition from traditional reactor plants to high-temperature nuclear reactors capable of operating at elevated coolant temperatures, and able to provide a large resource in terms of productivity. In thermonuclear energy, the emphasis is on the use of new types of nuclear fuel, the most promising of which is tritium. The use of tritium makes it possible to obtain a sufficient quantity of energy that can meet a number of needs in the energy sector and facilitate the abandonment of traditional energy sources [4,5,6]. Moreover, thermonuclear energy and power plants are among the most promising plants of the future. Research in this direction is both very relevant and timely since there are a large number of unresolved issues associated with their use.

One of the most important problems in thermonuclear energy is the problem of the production of tritium, which is an integral part of the fuel cycle of thermonuclear plants since it is used as the main nuclear fuel [7,8,9]. This problem is related to the low capacity for tritium production by traditional methods, which are not able to cover all the needs of thermonuclear installations. In this regard, much attention has been paid to the use of blanket materials consisting of lithium ceramics. The use of lithium ceramics based on titanates, silicates, or zirconates is potentially attractive because of the possibility of producing tritium as a result of nuclear reactions of lithium with neutrons. Moreover, most experts in this field agree that the use of lithium or lithium-containing ceramics will completely solve the problem of tritium fuel for thermonuclear energy, since lithium reserves will last for quite a long time [10,11,12]. The creation of blankets based on lithium ceramics must meet a number of requirements, the most significant of which are high mechanical strength and resistance to cracking and destruction under prolonged irradiation or temperature changes, excellent thermal conductivity, and increased resistance to thermal expansion (linear and volumetric) [13,14,15,16,17,18]. Of the currently known lithium ceramics, it is possible to single out ceramics based on lithium metazirconate, which, on account of the properties of zirconium oxide, not only have a higher resistance to destruction under external influences, including radiation embrittlement, but also thermophysical parameters that make it possible to influence the modes of use and service life [17,18].

The main hypothesis of this study concerns the assessment of the prospects of using a magnesium oxide (MgO) dopant [19,20], which is one of the most commonly used oxide compounds to increase the resistance of ceramics to external influences and phase transformation processes, and to maintain the long-term stability of the material properties during operation, for the modification of lithium-containing Li_2_ZrO_3_ ceramics, which have great potential as blanket materials for tritium propagation. The most pronounced advantages of Li_2_ZrO_3_ ceramics include high compatibility with structural materials and reflector materials, in particular with beryllium, high mechanical and corrosion resistance, and excellent tritium release and accumulation during irradiation [21,22,23,24]. Moreover, the doping of lithium-containing ceramics based on lithium metazirconates with magnesium oxide should make it possible to increase the resistance of ceramics to external influences, including mechanical strength and crack resistance, as well as increase in thermal conductivity. The tested hypothesis is based on assumptions regarding the properties of magnesium oxide, which enable it to form complex-phase ceramics because of the substitution or interstitial effect, which, in turn, can lead to the formation of interfacial boundaries and a rise in the dislocation density, which contributes to hardening [22,23,24]. If there is successful implementation of the technology for obtaining these ceramics, which are highly resistant to external influences, these ceramics can become one of the promising alternative materials for the production of tritium and hydrogen, which, in turn, will solve a number of issues in the energy sector with respect to the search for alternative energy sources.

## 2. Materials and Methods

The ceramics were synthesized by mixing the initial components in a planetary mill in order to achieve a homogeneous powder, which was subsequently subjected to thermal annealing. Under the selected grinding conditions, the processes of mechanochemical phase formation cannot be initiated. In this case, grinding was necessary to create homogeneously mixed powders of the initial components, which were subsequently annealed at a temperature of 1300 °C, at which the process of phase transformations took place. Powders of LiClO_4_ × 3H_2_O, ZrO_2_, and MgO in given stoichiometric ratios were used as the initial components for synthesis; the chemical purity was 99.95%. The MgO dopant concentration for the experiments ranged from 0.05 to 0.25 mol. Mixing was carried out using a planetary mill PULVERISETTE 6 classic line (Fritsch, Berlin, Germany) at a grinding speed of 400 rpm for 30 min. After grinding, the resulting mixtures were subjected to thermal sintering in a muffle furnace at a temperature of 1300 °C for 8 h, followed by cooling for 24 h. Annealing was conducted in a RUS-universal muffle furnace (RUS-universal, Moscow, Russia); the heating rate was 10 °C/min, the temperature was controlled using thermocouples, SiC heating elements were used as heating elements. The formulas of the chemical reactions expected during the synthesis after thermal annealing are shown below:

4LiClO_4_ + 2ZrO_2_ → 2Li_2_ZrO_3_ + 2Cl_2_ +7O_2_ (1)

Li_2_ZrO_3_ + MgO → MgLi_2_ZrO_4_ (2)

Using a priori data on the initial components used to obtain the ceramics, the Gibbs free energy (∆*G*) was calculated for both reactions that occur during the thermal annealing of the ground components. Values of ∆*G* = −15.6 kJ/mol for chemical reaction (1) and ∆*G* = −7.3 kJ/mol for reaction (2) were obtained. The negative value of ∆*G* for both reactions indicates that the formation of both Li_2_ZrO_3_ and MgLi_2_ZrO_4_ phases under the chosen temperature annealing conditions can occur freely.

An analysis of the variation in the phase composition of ceramics with variation in the dopant concentration, along with an assessment of the structural features and the structural ordering degree, was performed using the X-ray diffraction method. The diffraction patterns were acquired in the Bragg–Brentano geometry on a D8 Advance ECO powder diffractometer (Bruker, Berlin, Germany). The phase composition was refined using the DiffracEVA v.4.2 program code. Determination of the phase composition and the phase ratio was performed using card values from the PDF-2 (2016) database. Refinement of the crystal lattice parameters and the structural ordering degree was conducted using the method of comparative estimation of the deviation of the position of diffraction peaks from the reference values, followed by calculation of the experimentally acquired values of the parameters. The structural ordering degree was estimated from the change in the ratio of the crystalline phase contributions, characterized by the areas of the reflections, and the amorphous inclusions, which were described by approximating the area in the diffraction patterns characteristic of the background radiation.

The determination of porosity via the X-ray diffraction method was based on the following assumptions: One of the important parameters affecting the change in the density of materials is the crystal lattice volume, the alteration of which results in changes in the material density. Formula (3) was used to estimate the density [25]:

(3) $$p=\frac{1.6602\sum AZ}{V_{0}},$$

where *V*_0_ is the unit cell volume, *Z* is the number of atoms in a crystal cell, and *A* is the atomic weight of the atoms. Moreover, in the case of variation in the phase composition attributable to a change in the content of the phases, the density is calculated considering the contribution of each phase to the composition of the sample, and, as a result, the average density value calculated, taking into account the contribution factors of each phase, is used in further analyses.

The classical approach to determining the value of the porosity using X-ray diffraction methods is presented in the work of J.I. Goldstein [25], which describes exactly how to apply the X-ray diffraction method to determine porosity. Also, in the work of K. Meyer et al. [26], a detailed diagram of the applicability of various analysis methods is provided for determining the porosity, according to which X-ray and neutron diffraction methods are applicable in the pore size range from fractions of nanometers to 100–300 nm, which agrees closely with the pore sizes observed in the studied ceramic samples. The use of X-ray diffraction techniques for the characterization of porous materials is also presented in [27,28]. The porosity (*P_dil_*) is calculated using Formula (4) [29,30]:

(4) Pdil=(1−pp0)∗100%

where *p*_0_ is the density of the reference sample; as a rule, this value is taken from the PDF-2 database, which is typical for the given phase.

Impedance spectroscopy was conducted on a HIOKU IM3533-01 RLC-meter (HIOKU, Singapore) in the frequency range 2–200,000 Hz. The ceramic powders were pressed into tablets using polyvinyl alcohol as a binder. Silver paste was applied to both sides of the tablets to create electrical contacts.

The morphological features of the synthesized lithium-containing ceramics were studied by scanning electron microscopy using a Hitachi TM3030 scanning electron microscope (Hitachi, Tokyo, Japan).

Measurement of the strength properties of the lithium-containing ceramics doped with magnesium oxide depending on the dopant concentration was performed using the microindentation method using a LECO LM700 microhardness tester (LECO, Tokyo, Japan).

## 3. Results and Discussion

**Figure 1:**
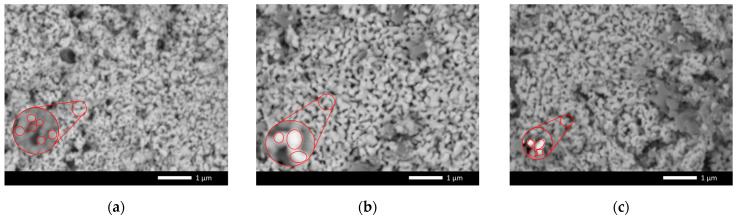

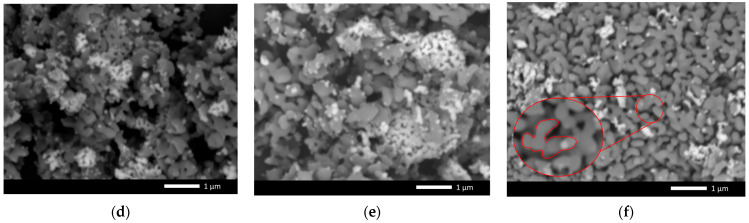
SEM images of synthesized xLi_2_ZrO_3_-(1-x)MgO ceramics versus MgO dopant concentration: (**a**) Pristine; (**b**) 0.05 mol; (**c**) 0.10 mol; (**d**) 0.15 mol; (**e**) 0.20 mol; (**f**) 0.25 mol. (red circles highlight the presence of fine grains characteristic of impurity inclusions of various phases).

Figure 1 demonstrates the results of morphological studies of the studied ceramics depending on the dopant concentration, reflecting the dynamics of grain size changes, with alteration in the ratio of the components in the composition of the ceramics. The presented SEM images generally indicate that the ceramics resulting after mechanical grinding and subsequent thermal annealing were ultradispersed nanometer-sized powders of spherical or oval shape (see inset in Figure 1a). It should be noted that a detailed analysis of the samples obtained after thermal annealing showed the presence of a porous surface, which indicates low strength of the ceramics.

When MgO was added to the initial mixture at concentrations of 0.05–0.10 mol, the formation of a fraction of larger particles having an oval or rhomboid shape was observed, and these particles were larger than the volume of the ceramics (see inset in Figure 1b,c).

At MgO dopant concentrations of 0.15 mol and higher, a sharp change in the morphology of the acquired particles was observed, with enlargement and subsequent formation of dendrites consisting of several particles (see insets in Figure 1f). Such a change in the morphological features may be associated with the effects attributable to a change in the phase composition of the ceramics during alloying, as well as the formation of new phases or a substitutional solid solution.

Using the ImageJ software code, the obtained images were analyzed in order to determine the particle sizes, as well as their variation with change in the dopant concentration. According to the data obtained from evaluating the average particle sizes, it was found that, in the case of the original (undoped) ceramics, the size was 50–100 nm; at dopant concentrations of 0.05–0.10 mol, the average size was no more than 130–150 nm, and the degree of homogeneity was more than 90%. In the case of dopant concentration above 0.15 mol, the average particle size was 200–250 nm, and change in the particle shape was observed. It should also be noted that, at dopant concentrations of 0.20–0.25 mol, a small quantity of fine grains was observed, the size of which was less than 30–50 nm.

**Figure 2:**
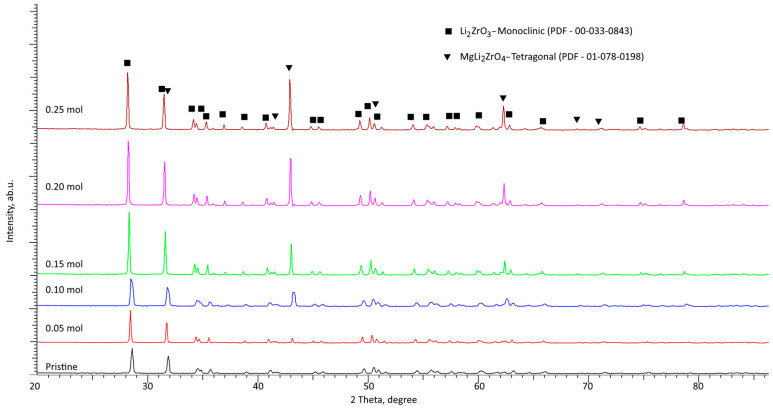
Results of X-ray diffraction of the studied xLi_2_ZrO_3_-(1-x)MgO ceramics versus MgO dopant concentration.

Determination of structural changes in xLi_2_ZrO_3_-(1-x)MgO ceramics depending on the concentration of the dopant, along with related phase transformations and processes of formation of the impurity phases, was conducted using the method of X-ray diffraction analysis. Figure 2 demonstrates the results of X-ray diffraction of the studied samples depending on the concentration of the dopant, reflecting changes in the phase composition of the ceramics. According to the presented data, thermal annealing of the acquired mixtures in the initial, undoped state resulted in the formation of a Li_2_ZrO_3_ structure with a monoclinic type of crystal lattice (spatial syngony C2/c(15)) and crystal lattice parameters a = 5.4021 Å, b = 9.0079 Å, c = 5.4110 Å, β = 112.31° (reference data of crystal lattice parameters a = 5.4266 Å, b = 9.0310 Å, c = 5.4227 Å, β = 112.72° for Li_2_ZrO_3_ PDF-00-033-0843). The differences in the parameters of the crystal lattice of the experimental sample from the reference values are associated with deformation processes caused by the grinding processes in a planetary mill, accompanied by strong deformation of the structure, substitution processes, and subsequent thermal annealing and relaxation of the deformation distortions. The degree of structural ordering of the undoped sample was 87.1%, which is typical for highly structurally ordered materials.

With respect to MgO dopant addition to the ceramic composition at a concentration of 0.05 mol, the creation of impurity inclusions or new phases was not identified, which points to the absence of phase formation or phase transformation processes at a given dopant concentration. The main changes observed for this sample were linked to an alteration in the parameters of the crystal lattice and elevation in the structural ordering degree to 88.5%, which indicated a decline in the defective fraction in the ceramic composition.

At MgO dopant concentrations of 0.10 mol and higher, the presented X-ray diffraction patterns had the appearance of diffraction reflections characteristic of the MgLi_2_ZrO_4_ (PDF-01-078-0198) tetragonal phase, the appearance of which was because of the processes of phase transformation and the formation of substitutional or interstitial solid solution phases related to them. Moreover, elevation in the MgO dopant concentration resulted in a rise in the contribution of the new phase in the composition of the ceramics, and, at a dopant concentration of 0.25 mol, resulted in insignificant dominance of MgLi_2_ZrO_4_ in the composition of the ceramics (more than 50%).

According to evaluation of the phase composition of the xLi_2_ZrO_3_-(1-x)MgO ceramics depending on MgO dopant concentration, it was found that a rise in the dopant concentration above 0.10 mol resulted in phase transformations of the Li_2_ZrO_3_ → Li_2_ZrO_3_/MgLi_2_ZrO_4_ type, followed by formation of two-phase ceramics with an equal content of the two phases.

**Figure 3:**
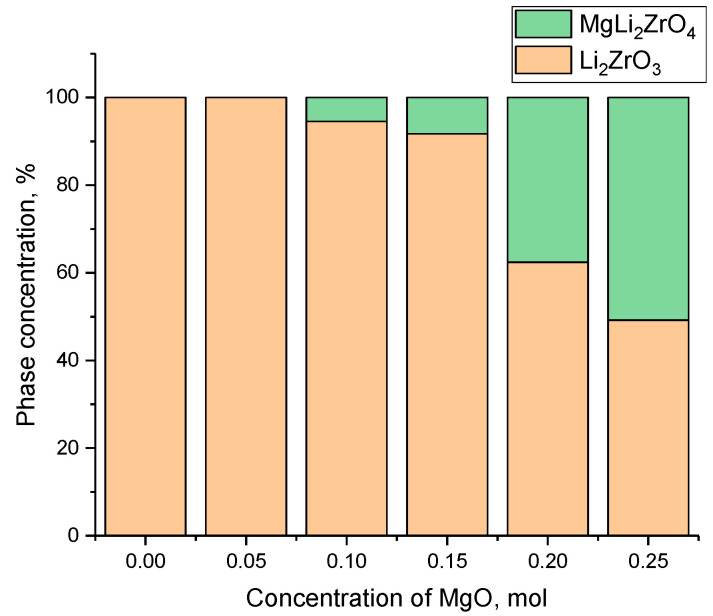
Variations in the phase composition of xLi_2_ZrO_3_-(1-x)MgO ceramics versus MgO dopant concentration.

Figure 3 demonstrates the results of estimating the variation in the phase composition depending on the concentration of the MgO dopant in the composition of the ceramics subjected to thermal annealing. Analysis of the phase composition, along with determination of the contributions of each phase, was conducted by calculating the areas for each phase and their ratio.

**Figure 4:**
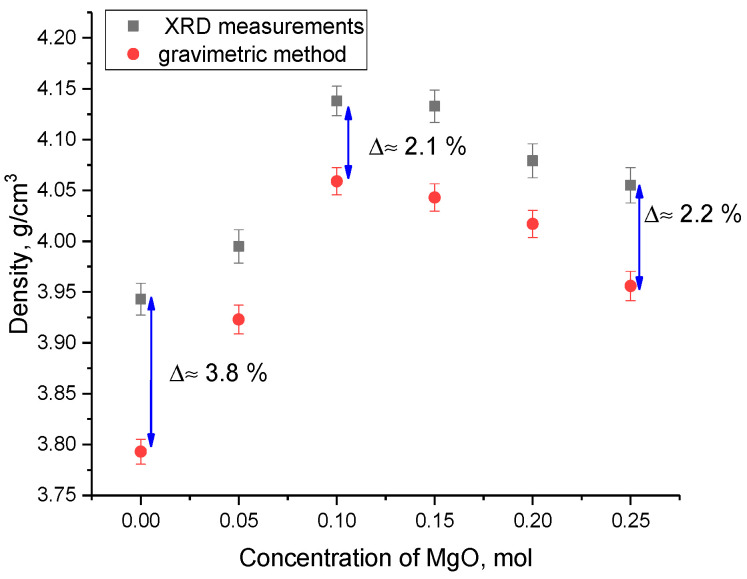
The assessment results of the xLi_2_ZrO_3_-(1-x)MgO ceramic density versus MgO dopant concentration.

As can be seen from the data presented in Figure 3, the main changes in the phase composition occurred at a MgO dopant concentration of 0.10 mol and were expressed, as shown in the X-ray diffraction patterns, in the appearance of characteristic reflections for the MgLi_2_ZrO_4_ tetragonal phase, increase in which was most pronounced at a MgO concentration of 0.20–0.25 mol. Based on the acquired data from the X-ray phase analysis, the crystal density was calculated; the results were then compared with the data acquired from the analysis of the density of the ceramics pressed into tablets using the gravimetric method (Archimedes method). The results of a comparative analysis of the change in the density of the ceramics depending on the MgO dopant concentration are shown in Figure 4. Density calculations using the X-ray phase analysis method were conducted considering changes in the parameters of the crystal lattice and its volume. The density of the ceramics was determined using two methods in order to establish their convergence. The classical gravimetric method (Archimedes method) was used to determine the density and porosity of the ceramics obtained in the form of tablets. The method for determination of the density of the ceramics based on the X-ray diffraction data consisted of the determination of changes in the crystal lattice volume and calculation of the effect of these changes on the material density. At the same time, according to the data obtained, there was good convergence between the two methods for determination of the density, and, as a result, the porosity.

As can be seen from the presented data, the change in the density of the xLi_2_ZrO_3_-(1-x) MgO ceramics depending on the MgO dopant concentration showed a pronounced maximum at concentrations of 0.10–0.15 mol, followed by a decline in density to 4.01–3.95 g/cm^3^ for dopant concentrations of 0.20–0.25 mol, respectively. The decrease in density for samples acquired at a dopant concentration of 0.20–0.25 mol was because of effects linked to the crystal lattice volume elevation.

**Figure 5:**
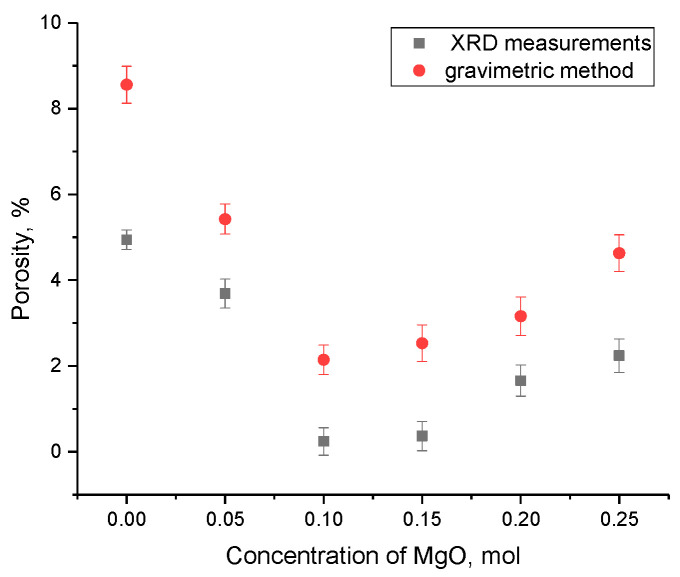
Results of changes in the porosity of xLi_2_ZrO_3_-(1-x)MgO ceramics versus MgO dopant concentration.

Figure 5 demonstrates the results of changes in the porosity of xLi_2_ZrO_3_-(1-x)MgO ceramics depending on the MgO dopant concentration.

Table 1 presents the data on the crystal lattice parameters of the established phases depending on the MgO dopant concentration in the composition of the ceramics. The calculation of the crystal lattice parameters was conducted by full-profile analysis with subsequent refinement of the parameters based on a comparative analysis of the position of the diffraction maxima with the given crystal lattice parameters for reference values from the PDF-2 (2016) database. The parameters were refined using card values for the Li_2_ZrO_3_ monoclinic phase (PDF-00-033-0843) and the MgLi_2_ZrO_4_ tetragonal phase (PDF-01-078-0198).

**Figure 6:**
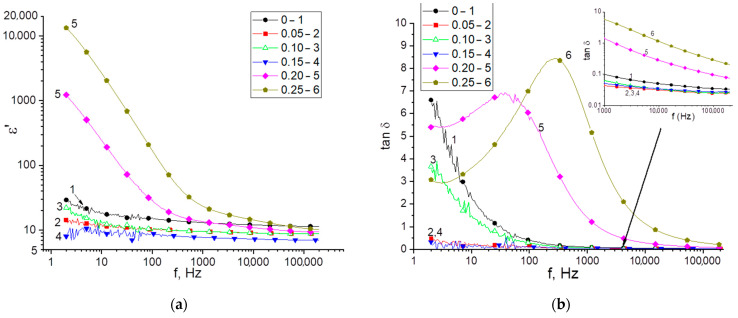
Frequency dependencies of the permittivity (**a**) and the dielectric loss tangent (**b**) of the acquired samples.

Figure 6 demonstrates the frequency dependencies of the dielectric constant and the tangent of the dielectric loss angle. The decrease in the permittivity with increasing frequency is linked to a delay in following the bound charges behind the alternating electric field where the mechanisms of thermal ionic and interfacial polarization cease to operate [31,32]. The pronounced maximum in the frequency spectra tan δ corresponds to the relaxation mechanism of polarization in the ceramics (Debye relaxation).

An analysis of the frequency dependencies demonstrates that, in different parts of the frequency range, the values of ε’, tan δ can differ significantly depending on the molar fraction of the MgO dopant. It is known that the value of the permittivity depends on the polarizability of the dipoles and the magnitude of the dipole moment, which, in crystals with ionic bonding, depends on the crystal structure. On the other hand, the permittivity is a structurally sensitive physical parameter. For this reason, the dielectric properties of ceramics are determined by two factors: the microstructure and the crystalline properties. In addition, at low frequencies, the permittivity value is strongly affected by through conduction caused by ion transport. Table 2 demonstrates the measurement results for the electrical conductivity, the permittivity and the dielectric loss tangent for some frequencies.

The increased electrical conductivity in samples with MgO 0.20 and 0.25 can be explained by the large grain size and the concomitant low defectiveness of the grain structure, which results in higher mobility of the charge carriers [33,34]. On the other hand, an increased value of σDC is linked to alteration in the phase composition of the ceramic. In the Li_2_ZrO_3_/MgLi_2_ZrO_4_ solid solution, Li^+^ cations can be replaced by Mg^2+^ cations, as a result of which the charge ratio between the crystal lattice sites can change. When Li^+^ is replaced by an ion with a higher oxidation state, Zr^4+^ → Zr^3+^ is oxidized to compensate for the charge ratio. In this case, an acceptor impurity can be created in the crystal grains, which generates an increased concentration of holes, which, in turn, increases the electrical conductivity [35]. As the dopant concentration increases, the proportion of the MgLi_2_ZrO_4_ phase increases, which means that the effective conductivity of the ceramic increases. The more pronounced dielectric properties of the ceramics (lower ε’, tan δ) at MgO fractions of 0.05 and 0.15 are related to the porosity, which can be detected in the SEM images. The volatilization of lithium upon annealing can also contribute to elevation in the permittivity in the high-frequency region because of the formation of charged defects that interact with the electric field. In general, the dielectric constant value of an undoped sample and samples with substitution have similar values in studies of the dielectric and structural properties of Li_2_ZrO_3_-MgO ceramics [36,37].

**Figure 7:**
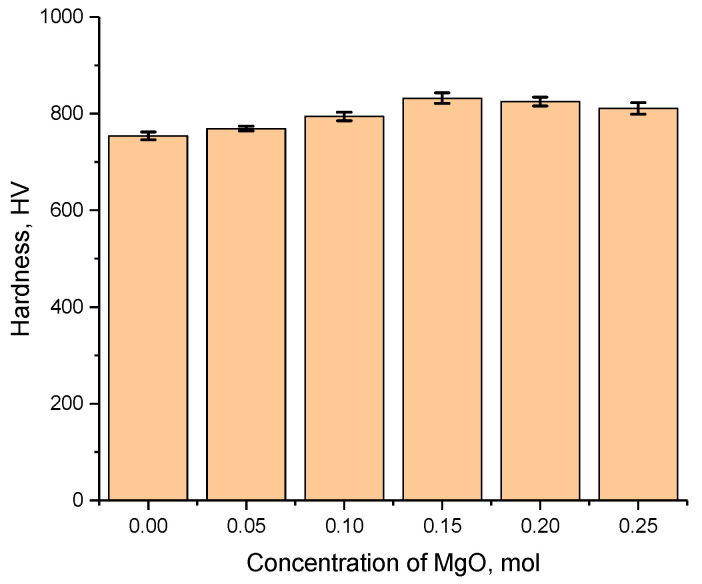
Variation in the hardness values of the studied ceramics versus MgO dopant concentration.

Analysis of the influence of changes in the strength properties of the synthesized ceramics depending on the MgO concentration was conducted by determining the indentation. A Vickers pyramid was used as an indenter; the load on the indenter was 100 N. The microhardness was determined by serial tests (25–30 indentations) from different parts of the ceramics, along with subsequent determination of the standard deviation and measurement error. Indentation in the form of serial tests was conducted to establish the isotropy of the strength properties of the ceramics, and to evaluate the influence of MgLi_2_ZrO_4_ phase formation on the resistance to external influences. The results of indentation are presented in Figure 7 as dependence of the change in hardness values on the MgO dopant concentration. Table 3 also presents the compressive strength and the flexible strength for the studied ceramics, which were calculated in order to determine the reliability and performance of materials under dynamic loading, as well as their tendency to brittle fracture resulting from external influences. The test was carried out on a special pendulum impact tester, according to ASTM D 7264/D7264M-07.

**Figure 8:**
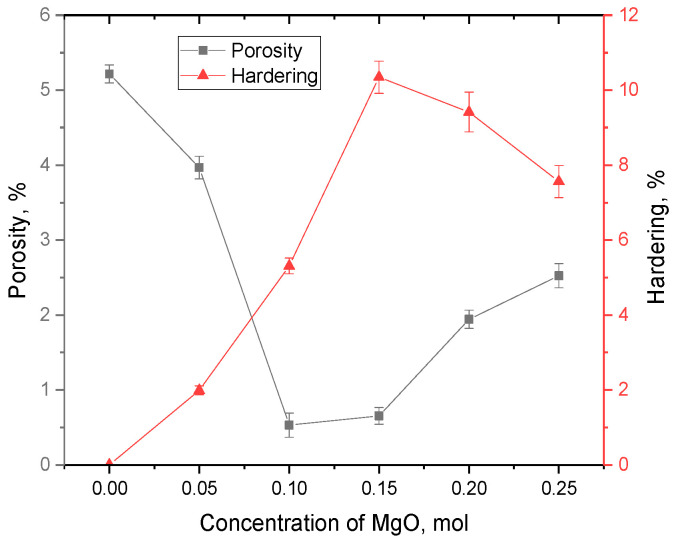
Variations in porosity and hardening versus MgO dopant concentration.

The overall appearance of the dependence of the change in the hardness of ceramics reflects the effect of hardening with a rise in the MgO dopant concentration at concentrations of 0.05–0.15 mol and a slight decrease in hardness compared to the maximum value at concentrations of the MgO dopant of 0.20–0.25 mol. The alteration in the hardness of the ceramics depending on the MgO dopant concentration was because of the effect of structural hardening and a rise in density as a result of a decline in the concentration of defective inclusions and a decline in the crystal lattice porosity. Moreover, analyzing the data on the changes in density and hardening presented in Figure 8, a correlation between the hardening of the ceramics and changes in the structural properties of the ceramics is clearly visible. The porosity was calculated from changes in the density of the ceramics compared to the reference density for the Li_2_ZrO_3_ (4.16–4.21 g/cm^3^) phase.

The hardening value was calculated based on a comparative analysis of changes in the hardness of the doped samples at different dopant concentrations with the original, undoped samples. The maximum increase in hardening was 10.3% for a sample with a dopant concentration of 0.15 mol compared to an undoped sample. In this case, hardening may be due not only to phase formation effects, but also to change in the porosity of the ceramics in the case of their compaction and change in density.

A further increase in concentration resulted in a decline in hardness by 1.0–2.0% in comparison with the maximum value, and, in comparison with the undoped sample, the hardening value exceeded 7–10%. Such an alteration in the strength properties, in addition to an alteration in the density and, as a consequence, the porosity of the crystal structure, can be explained by the formation of a two-phase structure of the ceramics at dopant concentrations above 0.10 mol.

**Figure 9:**
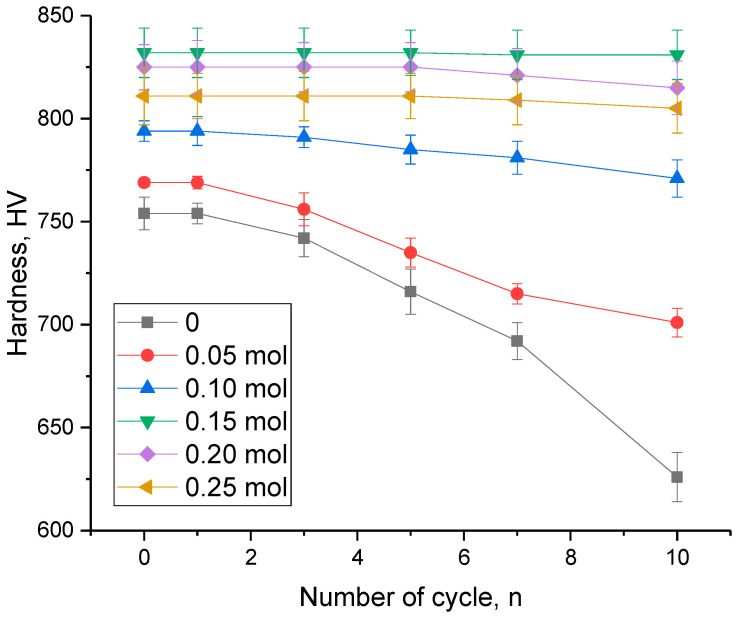
Evaluation results of changes in the hardness of ceramic specimens versus number of serial test cycles.

One of the key criteria for the applicability of ceramic materials as structural materials used under extreme conditions, including thermal heating, corrosion, and mechanical pressure, is the retention of their strength properties under prolonged external influences. Determination of resistance to high-temperature degradation and stress resistance during heating was conducted by conducting cyclic tests of samples when heated to 1000 °C, held at this temperature for 1 h and rapidly cooled, with the procedure repeated for several cycles. The maximum number of test cycles was 10 cycles. After 1, 3, 5, 7, and 10 cycles, the hardness values of the ceramic samples with different concentrations of the MgO dopant were measured. The results are presented in Figure 9 as dependence of the change in the hardness of the test samples on the number of test cycles.

As can be seen from the data presented, the general appearance of the observed changes indicates high-temperature degradation of the strength properties of the studied samples depending on the cyclic tests in the case of elevation in the number of cycles. Moreover, it should be noted that the trends in the change in the values of hardness, indicating strength disordering, are different in nature depending on the MgO dopant concentration in the composition of the ceramics. In the case of an undoped sample of ceramics, the decrease in hardness during the cyclic tests occurred already after three successive cycles, and the nature of the decrease in strength with a rise in the number of cycles of stress tests had a clear appearance of softening growth. Such behavior of specimens during endurance tests for stability may be because of the effects of high-temperature structural degradation during rapid heating and cooling of the specimens, which results in the appearance of a gradient of temperature jumps in the specimens related to alteration in the magnitudes of the thermal fluctuations. This results in destructive disordering of the crystal structure, which has a negative effect on the strength properties of the ceramics. With alteration in the structural ordering degree in the case of the addition of low concentrations of the MgO dopant, which results in densification of the ceramics, with a decline in hardness because of high-temperature degradation during the stress tests, a similar trend towards a decline in hardness is observed; however, the magnitude of the changes is slightly different in nature in comparison to the undoped ceramic samples.

According to the data acquired from the cyclic tests, formation of the MgLi_2_ZrO_4_ phase in the composition of the ceramics resulted in a rise in the resistance to thermal degradation in the case of the stress tests, which was expressed in the fact that the most noticeable decrease in hardness values was observed after 7–10 cyclic tests. This stability behavior indicates elevated resistance of the ceramics to thermally induced structural degradation. Moreover, it should be noted that the ceramics with a MgO dopant concentration of 0.15 mol had the highest stability, with the highest strength and low porosity observed. In the case of the doped ceramics, not only was a general increase in resistance to a destructive decrease in strength observed, but also a destruction rate decrease, which was expressed in the fact that the decline in hardness occurred after 5–7 cycles, while for the undoped ceramics, a similar decrease was already observed after 3 successive test cycles.

**Figure 10:**
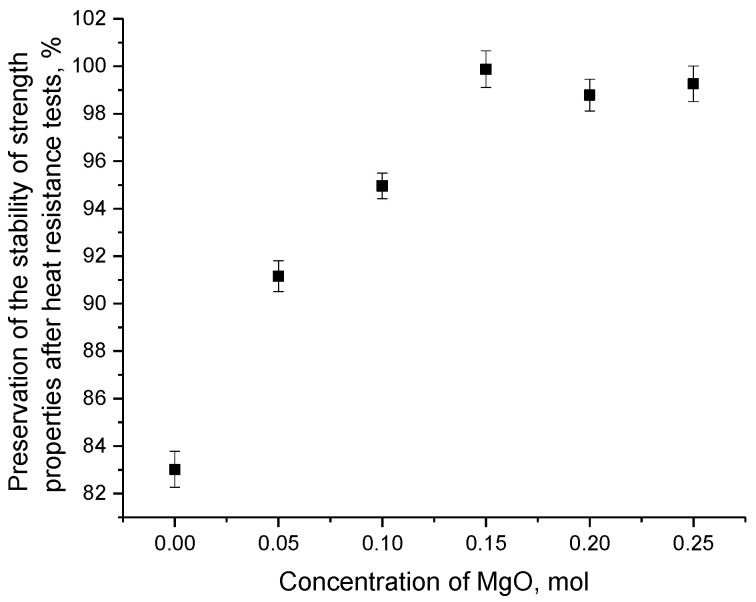
Results of maintaining the stability of strength properties after heat resistance tests.

An analysis of the acquired data on changes in the stability of the strength properties of the ceramics in the case of the thermal tests made it possible to estimate the strength degradation degree after the cyclic tests. The results of the degradation assessment are shown in Figure 10. Determination of the stability value was conducted by comparative analysis of the change in the hardness value of the ceramics before the thermal stability tests and after 10 successive test cycles.

As can be seen from the presented data on the change in the value of the stability of the strength properties of the ceramics after 10 successive cycles of testing for thermal stability, the ceramics in which the presence of inclusions in the form of the MgLi_2_ZrO_4_ phase was observed, because of a rise in the concentration of MgO in the composition of the ceramics, had the highest stability. In this case, hardening was primarily because of the formation of interfacial boundaries, the appearance of which resulted in alteration in the dislocation density, along with the density of the boundary effects. With respect to the single-phase Li_2_ZrO_3_ ceramics with a monoclinic type of crystal lattice, after 10 cycles of thermal tests, the decrease in hardness values was more than 15%, which indicates significant destruction of the near-surface layer, along with a decline in the crack resistance of the ceramics. The degradation in the strength properties as a result of the heat resistance tests was primarily because of the appearance of structurally distorted areas caused by alteration in the thermal vibrations of the crystal lattice, leading to destructive thermal expansion of the crystal structure of the ceramics and possible penetration of oxygen into the near-surface layer of the ceramics, with subsequent penetration into the nodes and interstices of the crystal lattice. In this case, the introduction of oxygen into the interstices can also lead to a partial expansion of the crystal lattice and growth in its volume, which results in a destructive change in the strength properties of the ceramics. Also, in the case of the undoped ceramics, the decrease in resistance to the thermal tests was because of the effects of high porosity and low density, which, in turn, resulted in a high probability of oxygen penetration into the ceramic composition, with possible subsequent introduction into the composition.

The addition of MgO to the composition of ceramics, which resulted in the formation of an MgLi_2_ZrO_4_ phase, along with subsequent increase in its contribution, resulted in the appearance of additional boundary effects and interfacial boundaries, which created additional obstacles to the introduction of oxygen, along with thermal expansion of the crystal lattice. Thus, the formation of the two-phase ceramics resulted in a rise not only in strength properties, but also in elevation in their resistance to the thermal tests and structural degradation under temperature differences. The overall change in strength properties for the two-phase ceramics after 10 successive cyclic tests was less than 1%.

## 4. Conclusions

In this paper, single- and two-phase Li_2_ZrO_3_-MgLi_2_ZrO_4_ ceramics with a high degree of structural order were obtained. Moreover, during the studies, it was found that the formation of the MgLi_2_ZrO_4_ impurity phase in the composition of the ceramics resulted in elevation in the hardness of the ceramics by 3–7%, which indicates a positive effect of the dopant related to hardening. Also, during the studies, it was found that the appearance of the MgLi_2_ZrO_4_ phase was accompanied by a rise in the density of the ceramics; however, at a high concentration of this phase, a slight decrease in density was observed. During tests for resistance to high-temperature heating, it was found that the formation of the MgLi_2_ZrO_4_ impurity phase resulted in the preservation of the stability of the strength characteristics, along with their resistance to destruction.

## Figures and Tables

**Table 1:** Crystal lattice parameter data.

Phase	MgO Dopant Concentration, mol. %					
	0	0.05	0.10	0.15	0.20	0.25
	Crystal Lattice Parameter, Å					
Li_2_ZrO_3_—Monoclinic	a = 5.40213, b = 9.00798, c = 5.41100, β = 112.300°	a = 5.39683, b = 8.97442, c = 5.40782, β = 112.014°	a = 5.38731, b = 8.95506, c = 5.40350, β = 111.924°	a = 5.40315, b = 8.95682, c = 5.40032, β = 111.814°	a = 5.34150, b = 8.99155, c = 5.41620, β = 111.968°	a = 5.41370, b = 8.93224, c = 5.40877, β = 112.034°
MgLi_2_ZrO_4_—Tetragonal	–	–	a = 4.18011, c = 9.12528	a = 4.19076, c = 9.14854	a = 4.19076, c = 9.14467	a = 4.20637, c = 9.15750

**Table 2:** Results of measurements of electrical conductivity σDC at direct current, dielectric constant ε’, and dielectric loss tangent at frequencies of 10 Hz, 100,000 Hz.

MgO Content, mol	σDC, S/m	ε’ (10 Hz)	ε’ (100,000 Hz)	tan δ (10 Hz)	tan δ (100,000 Hz)
0.00	6.12 × 10^−9^	18.26	11.48	2.29	0.0347
0.05	3.18 × 10^−10^	12.03	8.78	0.21	0.0248
0.10	2.94 × 10^−9^	13.04	8.76	1.49	0.0255
0.15	1.50 × 10^−10^	9.32	7.03	0.13	0.0272
0.20	2.15 × 10^−7^	243.73	9.47	6.02	0.0959
0.25	1.50 × 10^−6^	2651.26	10.80	3.60	0.2875

**Table 3:** Data of the compressive strength and the flexible strength.

Parameter	MgO Dopant Concentration, mol					
	0	0.05	0.10	0.15	0.20	0.25
The compressive strength, kJ/mm^2^	0.91 ± 0.05	1.02 ± 0.07	1.16 ± 0.11	1.25 ± 0.09	1.24 ± 0.08	1.21 ± 0.07
The flexible strength, MPa	142 ± 7	146 ± 8	163 ± 11	174 ± 12	169 ± 9	165 ± 6

## Data Availability

Not applicable.
